# Mechanisms of adverse effects of anti-VEGF therapy for cancer

**DOI:** 10.1038/sj.bjc.6603813

**Published:** 2007-05-22

**Authors:** T Kamba, D M McDonald

**Affiliations:** 1 Department of Urology, Kyoto University, 54 Kawahara-cho, Shogoin, Sakyo-ku, Kyoto 606-8507, Japan; 2 Comprehensive Cancer Center and Cardiovascular Research Institute, University of California San Francisco, 513 Parnassus Avenue, San Francisco, CA 94143-0452, USA; 3 Department of Anatomy, University of California San Francisco, 513 Parnassus Avenue, San Francisco, CA 94143-0452, USA

**Keywords:** angiogenesis inhibitors, VEGF signalling, capillaries

## Abstract

Advances in understanding the role of vascular endothelial growth factor (VEGF) in normal physiology are giving insight into the basis of adverse effects attributed to the use of VEGF inhibitors in clinical oncology. These effects are typically downstream consequences of suppression of cellular signalling pathways important in the regulation and maintenance of the microvasculature. Downregulation of these pathways in normal organs can lead to vascular disturbances and even regression of blood vessels, which could be intensified by concurrent pathological conditions. These changes are generally manageable and pose less risk than the tumours being treated, but they highlight the properties shared by tumour vessels and the vasculature of normal organs.

Proliferation of new blood vessels is necessary for tumours to grow and contributes to the spread of blood-borne metastases (Folkman, 1971). Vascular endothelial growth factor (VEGF) not only drives angiogenesis, but also serves as a survival factor for endothelial cells and promotes the abnormal phenotype of blood vessels in tumours (Inai *et al*, 2004). Unlike tumour vessels that have VEGF as survival factor, the normal adult vasculature is regarded as largely independent of VEGF for survival, stability, and normal function (Longo *et al*, 2002). Indeed, the rationale for using VEGF inhibitors on tumours is based on the assumption that tumour vessels can be impacted without harming other vessels. Consistent with this assumption, clinical studies have demonstrated that VEGF inhibitors have robust actions on certain types of tumours with infrequent serious side events (Herbst, 2006; Hurwitz and Saini, 2006).

This review examines current views of the basis of adverse events associated with VEGF inhibitors in the treatment of cancer. Many agents that inhibit VEGF signalling are under development, in clinical trials, or approved for use in cancer (http://www.cancer.gov/clinicaltrials/developments/anti-angio-table). Rapidly expanding use of these agents in the clinic reflects their efficacy. Increased use is also advancing the understanding of their actions in tumours and increasing familiarity with the adverse effects associated with their use (Hurwitz and Saini, 2006). Adverse events in cancer therapy include any unfavourable symptom, sign, laboratory finding, or disease temporally associated with the use of a medical treatment that may or may not be related to the treatment (Common Toxicity Criteria Manual (NCI-CTC), Cancer Therapy Evaluation Program, National Cancer Institute, U.S. National Institutes of Health, http://ctep.cancer.gov/. Adverse events are graded as absent (Grade 0), mild (Grade 1), moderate (Grade 2), severe and undesirable (Grade 3), life-threatening or disabling (Grade 4), or fatal (Grade 5)). Tumour progression or signs and symptoms directly related to the tumour are not considered as adverse events in this context.

## ANTI-VEGF AGENTS IN CLINICAL USE

Three drugs, bevacizumab (Avastin®), sunitinib malate (Sutent®, SU11248), and sorafenib (Nexavar®, BAY 43-9006) that were developed for antiangiogenic actions have been approved by the United States Food and Drug Administration (FDA) for treatment of patients with specific types of cancer. All three agents inhibit VEGF signalling by blocking VEGF ligand or VEGF receptor function. Sunitinib and sorafenib inhibit platelet-derived growth factor receptors (PDGFR) and some other receptor tyrosine kinases as well.

### Bevacizumab

Bevacizumab is a humanised, function-blocking monoclonal antibody that selectively binds VEGF. Bevacizumab is the first VEGF inhibitor approved by the FDA for systemic use in cancer. The anti-VEGF antibody is currently approved in combination with intravenous 5-fluorouracil (5-FU)-based chemotherapy, which typically is a combination of irinotecan, 5-FU, and leucovorin (IFL), for first- or second-line treatment of metastatic carcinoma of the colon or rectum (Hurwitz *et al*, 2004; Hurwitz and Saini, 2006) and, in conjunction with paclitaxel and carboplatin, for first-line treatment of unresectable, locally advanced, recurrent or metastatic nonsquamous, non-small cell lung cancer (NSCLC) (Sandler *et al*, 2006). Bevacizumab also has activity in breast cancer and kidney cancer. Bevacizumab is generally safe and well tolerated but can be accompanied by a variety of adverse effects, which are broadened or intensified by concurrent chemotherapeutic agents (Gordon and Cunningham, 2005; Hurwitz and Saini, 2006). The most common are hypertension, proteinuria, epistaxis, upper respiratory infection, anorexia, stomatitis, diarrhoea or other gastrointestinal symptoms, headache, dyspnea, fatigue, and exfoliative dermatitis. Infrequent serious adverse events include gastrointestinal perforation, haemorrhage, arterial thromboembolic events, hypertensive crisis, wound healing complications, neutropenia, nephrotic syndrome, reversible posterior leukoencephalopathy syndrome (RPLS), and congestive heart failure (http://www.gene.com/gene/products/information/oncology/avastin/index.jsp).

### Sunitinib

Sunitinib is an orally administered, small molecule inhibitor of multiple receptor tyrosine kinases implicated in tumour growth, angiogenesis, and metastatic progression. Sunitinib inhibits phosphorylation of VEGF receptors (VEGFR-1, -2, -3), platelet derived growth factor receptors (PDGFR-*α* and -*β*), stem cell factor receptor (KIT), Fms-like tyrosine kinase-3 (FLT3), colony stimulating factor receptor Type 1, and glial cell-derived neurotrophic factor receptor. Sunitinib is approved for treatment of advanced renal cell carcinoma and gastrointestinal stromal tumours (GIST) after disease progression on or intolerance to imatinib mesylate (Gleevec®). Clinical trials of patients with anthracycline- and taxane-resistant breast cancer are evaluating sunitinib in combination with taxanes (paclitaxel and docetaxel) in the first-line setting, in combination with capecitabine in the second-line setting, and as a single agent for tumours lacking HER2 receptors, estrogen receptors, and progesterone receptors (http://www.clinicaltrials.gov/ct/show). Sunitinib is generally well tolerated. The most common adverse reactions, occurring in more than 20% of patients, are fatigue, asthenia, diarrhoea, nausea, mucositis/stomatitis, vomiting, dyspepsia, abdominal pain, constipation, hypertension, rash, hand-foot syndrome, skin discolouration, altered taste, anorexia, and mild bleeding (http://www.sutenthcp.com/prescribing_information.asp).

### Sorafenib

Sorafenib is an oral, small molecule inhibitor of multiple tyrosine kinase receptors involved both in angiogenesis and tumour cell proliferation: VEGFR-2, VEGFR-3, PDGFR-*β*, RAF kinase, FLT3, KIT, p38 MAP kinase (p38-alpha, MAPK14). Sorafenib is approved for treatment of advanced renal cell carcinoma and is in phase III clinical trials for hepatocellular carcinoma, metastatic melanoma, and NSCLC. Phase I/II trials of sorafenib plus chemotherapy are ongoing for other solid tumours (Morabito *et al*, 2006). Side effects associated with sorafenib are mostly mild to moderate, with few severe (Grade 3–4) toxicities. Rash, exfoliative dermatitis, hand-foot skin reaction, diarrhoea, and fatigue are the most common adverse events, occurring in 33–38% of patients, and are usually Grade 1 or 2. Mild hypertension, leukopenia, or bleeding is also common. Life-threatening haemorrhage, cardiac ischaemia or infarction, RPLS, and gastrointestinal perforation are uncommon (http://www.nexavar.com/wt/page/index).

## PRECLINICAL EVIDENCE OF EFFECTS OF VEGF INHIBITION ON THE NORMAL ADULT VASCULATURE

Preclinical studies of VEGF inhibitors are beginning to elucidate the mechanism of some adverse events found in the clinic. From one perspective, adverse effects of VEGF inhibitors may be considered consequences of blocking actions of VEGF in normal physiology. The essential role of VEGF during embryonic development is well established and widely accepted, but this dependency was thought not to persist into adult life. Yet, actions of VEGF are beginning to be identified in normal organs of the adult, examples being the role of VEGF in function and survival of normal blood vessels, blood pressure regulation, and renal, neurological, and hepatic function (Horowitz *et al*, 1997; Eremina *et al*, 2003; DeLeve *et al*, 2004; Kamba *et al*, 2006; Lambrechts and Carmeliet, 2006). Findings from studies of structural or functional changes in normal organs after inhibition of VEGF signalling provide clues into mechanisms of side effects in cancer patients treated with VEGF inhibitors.

**Figure 1:**
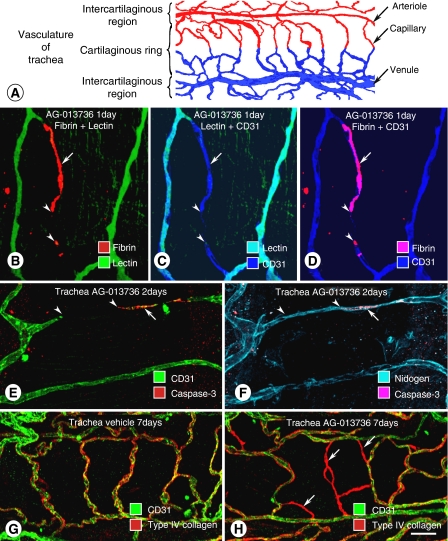
Simple vascular network of tracheal mucosa used to examine effects of VEGF inhibition on normal blood vessels in adult mice. (**A**) Tracheal vasculature has a simple, repetitive network of arterioles, capillaries, and venules aligned with each cartilaginous ring (Baffert *et al*, 2004). (**B**–**D**) Confocal microscopic images of tracheal capillaries showing deposits of fibrin in nonpatent segment of tracheal capillary after inhibition of VEGF signalling by AG-013736 for 1 day. Fibrin deposit (arrow) is shown to be in a nonperfused capillary segment by absence of lectin binding, and is near a region of capillary regression that lacks CD31 immunoreactivity (arrowheads) (Baffert *et al*, 2006b). (**E**–**F**) Confocal images of tracheal vasculature showing apoptotic endothelial cells stained for activated caspase-3 (arrow), near region of capillary regression (arrowheads) shown by absence of CD31 immunoreactivity (**E**). Vascular basement membrane persists after endothelial cells regress, as shown by uninterrupted nidogen immunoreactivity (**F**) (Baffert *et al*, 2004). (**G**–**H**) Confocal micrographs show colocalisation of CD31 (green) and type IV collagen (red) on normal vasculature (**G**). After AG-013736 for 7 days, empty sleeves of type IV collagen (red, arrows) replace some normal mucosal blood vessels (**H**) (Inai *et al*, 2004). Scale bar in (H): 20 *μ*m in (**B**–**D**); 25 *μ*m in (**E**) and (**F**); 30 *μ*m in (**G**) and (**H**).

Studies of the effects of pharmacologic inhibitors in mice indicate that VEGF participates in blood vessel survival and plasticity in adult life. Examination of the simple vascular network of the mouse trachea (Figure 1A), treated systemically for 1–28 days with an inhibitor of VEGF signalling, revealed rapid regression of some normal mucosal capillaries (Baffert *et al*, 2004, 2006a; Inai *et al*, 2004). After only 1 day of treatment, fibrin accumulated and patency was lost in some capillaries (Figure 1B–D; Baffert *et al*, 2004, 2006a; Inai *et al*, 2004). By 2 days, endothelial cells underwent apoptosis and regression. The magnitude of capillary loss after 10-day treatment depended on the age of the mice: 39% at 4 weeks of age, 28% at 8 weeks, and 14% at 16 weeks (Baffert *et al*, 2004). Empty sleeves of vascular basement membrane persisted for several weeks after endothelial cells regressed (Figure 1E and F), and not only marked the location of capillary regression, but also served as a scaffold for vascular regrowth (Figure 1G and H; Inai *et al*, 2004; Baffert *et al*, 2006a).

**Figure 2:**
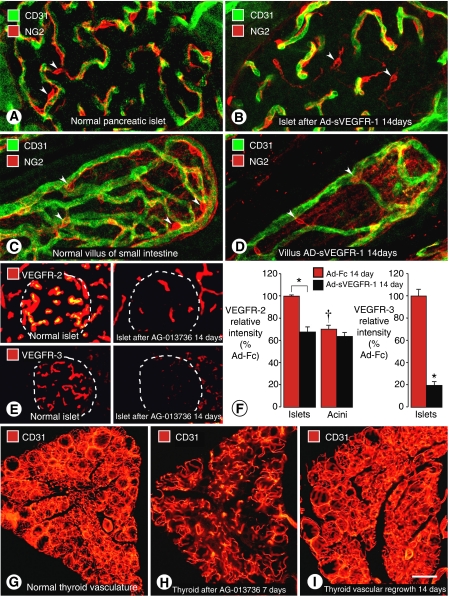
Regression of capillaries in vasculature of normal adult mice after inhibition of VEGF signalling. (**A**–**D**) Confocal microscopic images showing capillaries in pancreatic islets (**A** and **B**) and villi of small intestine (**C** and **D**) under baseline conditions and after VEGF inhibition. After Ad-sVEGFR-1 for 14 days, endothelial cells of some capillaries have regressed, leaving pericytes (red, NG2, arrowheads) at sites of regression (Kamba *et al*, 2006). (**E**) Comparison of VEGFR-2 and VEGFR-3 immunofluorescence in pancreatic islet capillaries after VEGF inhibition. Stronger endothelial cell VEGFR-2 immunoreactivity under baseline conditions (upper left) than after Ad-sVEGFR-1 for 14 days (upper right). Stronger endothelial cell VEGFR-3 immunoreactivity under baseline conditions (lower left) than after Ad-sVEGFR-1 for 14 days (lower right). (**F**) Bar graphs showing fluorescence intensities of VEGFR-2 and VEGFR-3 immunoreactivities under baseline conditions and after Ad-sVEGFR-1 for 14 days (Kamba *et al*, 2006). ^*^P<0.05, significantly different from corresponding control. †*P*<0.05, significantly different from islets. (**G**–**I**) Fluorescence micrographs of thyroid capillaries stained for CD31 immunoreactivity show dense vascularity under baseline conditions (**G**), loss of half of the capillaries after AG-013736 for 7 days (**H**), and complete regrowth of vasculature during 14 days after end of treatment (**I**) (Kamba *et al*, 2006). Scale bar in I: 25 *μ*m in (**A**–**D**); 40 *μ*m in (**E**) and (**F**); 160 *μ*m in (**G**–**I**).

A survey of 18 organs of normal adult mice revealed significant regression of capillaries in some organs and not in others (Kamba *et al*, 2006; Baffert *et al*, 2006a). After inhibition of VEGF signalling for 1 to 3 weeks, significant capillary regression occurred in pancreatic islets (Figure 2A and B), thyroid, adrenal cortex, pituitary, villi of small intestine (Figure 2C and D), choroid plexus, adipose tissue, and trachea (Kamba *et al*, 2006; Baffert *et al*, 2006a). The amount of regression was dose-dependent and varied from organ to organ, with a maximum of 68% in thyroid. But two tumours examined under the same conditions had even greater vascular regression (Inai *et al*, 2004; Kamba *et al*, 2006). Little or no capillary regression was detected in brain, retina, skeletal muscle, cardiac muscle, or lung under these conditions. Capillaries that underwent regression had the same pericyte coverage and apparent structural maturity as capillaries that survived.

A feature that distinguished capillaries that underwent regression from those that survived was expression of relatively high levels of VEGFR-2 and VEGFR-3 in endothelial cells (Figure 2E and F). Inhibition of VEGF signalling was accompanied by decreased expression of VEGFR-2 and VEGFR-3 in surviving capillaries (Figure 2E and F; Baffert *et al*, 2004; Kamba *et al*, 2006). Another distinguishing feature of VEGF-dependent capillaries was the presence of endothelial fenestrations. These 80–100-nm pores are a normal component of endothelial cells of endocrine organs, gastrointestinal tract, choroid plexus, and kidney (Kamba *et al*, 2006).

Studies of the reversibility of capillary regression after inhibition of VEGF signalling showed that, strikingly, most capillaries in the thyroid grew back within 1 or 2 weeks, even when 50–60% of capillaries had regressed during the 7-day treatment (Figure 2G–I; Mancuso *et al*, 2006). Tracheal capillaries also regrew (Baffert *et al*, 2006a). Rapid regrowth appears to be facilitated by empty sleeves of basement membrane and accompanying pericytes that provide a scaffold for revascularisation (Mancuso *et al*, 2006).

**Figure 3:**
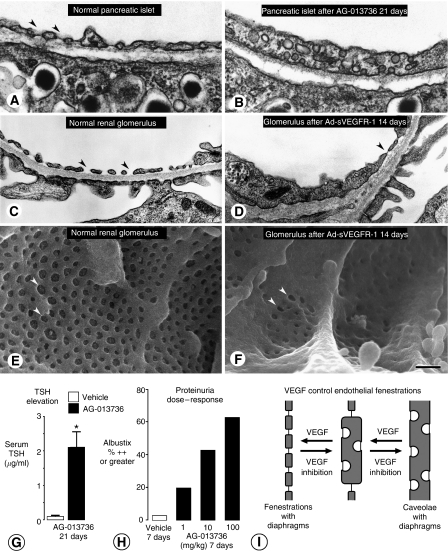
Reduction in endothelial fenestrations (arrowheads) after inhibition of VEGF signalling. (**A** and **B**) Transmission electron microscopic images of islet capillaries showing thin endothelium and abundant fenestrations with diaphragms under baseline conditions compared to thick endothelium, few fenestrations, and abundant caveolae after AG-013736 for 21 days (Kamba *et al*, 2006). (**C** and **D**) Transmission EM images of renal glomerular capillaries comparing thin endothelium and abundant fenestrations under baseline conditions with thick endothelium and few fenestrations after Ad-sVEGFR-1 for 14 days (Kamba *et al*, 2006). (**E** and **F**) Scanning electron microscopic images of luminal surface of glomerular capillaries showing abundant endothelial fenestrations under baseline conditions and few fenestrations after Ad-sVEGFR-1 for 14 days (Kamba *et al*, 2006). (**G**) Bar graph showing significantly higher concentration of TSH in serum as a measure of altered thyroid function after AG-013736 for 21 days. (**H**) Bar graph showing increasing amount of proteinuria, indicated by proportion of mice with Albustix values of ++ or greater (⩾100 mg albumin/dl of urine), with increasing dose of AG-013736 for 7 days. (**I**) Diagram of hypothetical shuttling of diaphragms between endothelial fenestrations and caveolae, with VEGF inhibition driving the process to the right and VEGF signalling driving it to the left (Kamba *et al*, 2006). Scale bars: 0.3 *μ*m in (**A**) and (**B**); 1 *μ*m in (**C**) and (**D**); 0.5 *μ*m in (**E**) and (**F**).

Reductions in endothelial fenestrations were evident within 24 h of inhibition of VEGF signalling (Inai *et al*, 2004). In the thyroid, which has heavily fenestrated capillaries, fenestrations were reduced by as much as 88% on capillaries that survived 7 days of treatment. Pancreatic islet capillaries showed a similar reduction in endothelial fenestrations (Figure 3A and B; Kamba *et al*, 2006). By comparison, skeletal muscle capillaries, which have no endothelial fenestrations, showed no significant reduction in number after treatment for 7 days (Inai *et al*, 2004). Endothelial fenestrations were also conspicuously reduced in the renal glomerulus, whether assessed by transmission electron microscopy (Figure 3C and D) or by scanning electron microscopy (Figure 3E and F; Kamba *et al*, 2006).

Mice treated with inhibitors of VEGF signalling did not lose weight and appeared healthy, but did have certain physiological changes. Among these were elevation of thyroid stimulating hormone (TSH), indicative of altered thyroid function (Figure 3G), and dose-related proteinuria (Figure 3H; Inai *et al*, 2004; Kamba *et al*, 2006).

Together, these preclinical findings indicate that many capillaries with endothelial fenestrations are dependent on VEGF signalling (Inai *et al*, 2004; Kamba *et al*, 2006). Changes in this population of blood vessels also reflect the dynamic nature of endothelial fenestrations (Figure 3I) and the potential plasticity of the microvasculature in some normal organs (Kamba *et al*, 2006). Further, the findings raise the possibility that tumours with fenestrated capillaries, such as those arising in endocrine glands or the gastrointestinal tract, are particularly sensitive to inhibitors of VEGF signalling. Presence of endothelial fenestrations in tumour vessels may reflect sensitivity to VEGF inhibition and help predict therapeutic response (Inai *et al*, 2004; Kamba *et al*, 2006).

## ASSESSMENT OF ADVERSE EFFECTS OF VEGF SIGNALLING INHIBITORS FROM PRECLINICAL AND CLINICAL PERSPECTIVES

### Hypertension

Hypertension is one of the best-documented and most frequently observed side effects of systemic inhibition of VEGF signalling. Hypertension, which can occur anytime after the initiation of treatment, usually can be managed with oral antihypertensive agents, and treatment can be continued without reduction in dose. In patients on bevacizumab, hypertension had an overall incidence of up to 32% (Hurwitz *et al*, 2004; Kabbinavar *et al*, 2005); 11–16% of patients required intensive therapy with multiple drugs (Grade 3), but only 1% had life-threatening hypertensive crisis (Grade 4). In patients on sunitinib, hypertension had an incidence of 28% (6% had Grade 3) in phase II trials in metastatic renal cell carcinoma (Motzer *et al*, 2006) and an incidence of 15% (4% had Grade 3) in a phase III trial in GIST (Demetri *et al*, 2005). In patients on sorafenib, hypertension had an overall incidence of 17% (3% had Grade 3 or 4) (Kane *et al*, 2006).

An important part of the mechanism of hypertension associated with VEGF inhibition is thought to involve decreased production of nitric oxide (NO) in the wall of arterioles and other resistance vessels. Vascular endothelial growth factor increases NO synthesis through upregulation of endothelial NO synthase, and VEGF inhibition diminishes NO synthesis (Horowitz *et al*, 1997; Hood *et al*, 1998). Because NO is a vasodilator, decreased NO synthesis promotes vasoconstriction, increased peripheral resistance, and increased blood pressure. Effects of VEGF inhibition on the control of blood pressure by the kidney may also be involved after prolonged treatment.

### Proteinuria

The common occurrence of proteinuria after inhibition of VEGF signalling reflects the importance of VEGF in normal renal function (Eremina *et al*, 2003; Schrijvers *et al*, 2004). Proteinuria was found in 23% of 1132 patients in clinical trials of bevacizumab in various types of cancer and was more common in patients receiving bevacizumab plus chemotherapy than in patients on chemotherapy alone (Hurwitz *et al*, 2004; Kabbinavar *et al*, 2005). Proteinuria is typically asymptomatic and decreases after treatment ends. Serious impairment of renal function is rare.

The filtration barrier of the renal glomerulus is formed by endothelial cells, basement membrane, and podocytes. Vascular endothelial growth factor, which is expressed by podocytes both during development and in the adult, activates VEGFR-2 on glomerular capillary endothelial cells. Targeted heterozygous deletion of VEGF in podocytes results in renal pathology manifested by loss of endothelial fenestrations in glomerular capillaries, proliferation of glomerular endothelial cells (endotheliosis), loss of podocytes, and proteinuria in mice (Eremina *et al*, 2003; Schrijvers *et al*, 2004). Pharmacological inhibition of VEGF signalling in mice also reduces endothelial fenestrations in glomerular capillaries (Kamba *et al*, 2006). Inhibition of VEGF-dependent interactions between podocytes and glomerular endothelial cells disrupts the filtration barrier, which in turn leads to dose-dependent proteinuria (Figure 3H; Eremina *et al*, 2003; Kamba *et al*, 2006).

### Impaired wound healing

Angiogenesis is a necessary step in wound healing (Bates and Jones, 2003). Agents that impair blood vessel growth might therefore be expected to interfere with wound repair. Wound healing was evaluated in patients who underwent surgery 0–60 days after the last dose of bevacizumab or 28–60 days before the first dose of bevacizumab in phase II and III trials in colorectal cancer (Scappaticci *et al*, 2005). Bevacizumab in serum has a half-life of about 20 days. Grade 3 or 4 complications included delayed or abnormal wound healing, wound dehiscence, bowel perforation, fistula, abscess, and haemorrhage. In patients who had already received bevacizumab, complications occurred in 10 of 75 patients (13%), when surgery was performed within 60 days of the last dose, but only 1 of 29 patients (3.4%) had complications when surgery followed chemotherapy alone. Another arm of the study found complications in 3 of 230 patients (1.3%), when surgery preceded bevacizumab and in 1 of 194 patients (0.5%) when surgery preceded chemotherapy alone.

Results of preclinical studies suggest that effects of inhibition of VEGF signalling are more complex than simple impairment of revascularisation (Ko *et al*, 2005). Wound strength, re-epithelialisation, and other factors may also be involved, and agents that block multiple targets may have different effects than agents that selectively inhibit VEGF (Ko *et al*, 2005). Shorter half-lives of sunitinib, sorafenib, and other small-molecule inhibitors would be expected to be accompanied by more rapid recovery of normal wound healing.

### Gastrointestinal perforation

Gastrointestinal perforation is an infrequent but potentially life-threatening event during anti-VEGF therapy (Hurwitz and Saini, 2006). In a phase III study of colorectal cancer, gastrointestinal perforation occurred in six patients (1.5%) in the bevacizumab/IFL arm, compared to none in the placebo/IFL arm (Hurwitz *et al*, 2004). One of the affected patients died and two discontinued therapy. Similarly, gastrointestinal perforation was found in 1.7% of 1968 patients receiving bevacizumab plus first-line chemotherapy for metastatic colorectal cancer in a community-based observational registry (Hedrick *et al*, 2006). Perforations occurred most commonly (68%) during the first 60 days of treatment. Gastrointestinal perforation occurs in less than 1% of patients on sorafenib. Perforation and fistula formation or peritonitis are also uncommon adverse events in patients treated with sunitinib for intra-abdominal malignancies. Risk factors include tumour at the site of perforation, abdominal carcinomatosis, acute diverticulitis, bowel obstruction, recent history of sigmoidoscopy or colonoscopy, and history of pelvic or abdominal irradiation.

Although the mechanism underlying gastrointestinal perforation is unknown, abscesses, diverticula, and sites of bowel resection and anastomosis have been implicated in some cases (Kabbinavar *et al*, 2005; Giantonio, 2006). In normal adult mice, inhibition of VEGF signalling for 2–3 weeks causes regression of 34–46% of capillaries of intestinal villi (Kamba *et al*, 2006). This change does not appreciably impair intestinal function as judged from maintenance of body weight, but could contribute to perforation in the presence of concurrent inflammation or other pathological conditions.

### Haemorrhage and thrombosis

Bleeding events, including epistaxis, haemoptysis, haematemesis, gastrointestinal bleeding, vaginal bleeding, and brain haemorrhage, are found in some patients on bevacizumab, sunitinib, or sorafenib. Mild epistaxis (Grade 1) is the most common form of haemorrhage found with bevacizumab and usually resolves without medical intervention. Mild epistaxis and other mild haemorrhagic events were found in 26% of patients receiving sunitinib for metastatic renal cell carcinoma. Rectal, gingival, upper gastrointestinal, genital, and wound bleeding were less common.

Grade 3–5 haemorrhage occurred with an incidence of 9% and mortality of 6% of patients treated with bevacizumab for NSCLC (Johnson *et al*, 2004; Sandler *et al*, 2006). When patients with squamous cell carcinoma were excluded from the phase II trial of bevacizumab, fatal haemoptysis had an incidence of 1% (5/420) (http://www.roche.com/inv-update-2005-05-16). Fatal pulmonary haemorrhage occurred in two patients in a trial of sunitinib in metastatic NSCLC (Socinski *et al*, 2006), and the agent is not approved for this form of cancer. Patients with metastatic colorectal cancer who receive bevacizumab plus chemotherapy generally have a higher incidence of serious haemorrhage (3–9%) than those on chemotherapy alone (Kabbinavar *et al*, 2003; Hurwitz *et al*, 2004; Giantonio *et al*, 2005; Gordon and Cunningham, 2005; Kabbinavar *et al*, 2005; Hurwitz and Saini, 2006) (Other information at: http://www.avastin-info.com/portal/eipf/pb/avastin/com/clinicalslidekit). Haemorrhagic events of all types had a higher incidence in patients on sorafenib (15%) than on placebo (8%). No difference in haemorrhagic events was found between patients with GIST receiving sunitinib (18%) and those receiving placebo (17%).

Treatment with bevacizumab is also accompanied by increased risk of arterial thromboembolic events, including stroke, transient ischaemic attack, subarachnoid haemorrhage, myocardial infarction, and angina, particularly in patients over 65 years of age or with a history of thromboembolic events. In a pooled analysis of data from five trials in colorectal cancer, NSCLC, and metastatic breast cancer, the overall incidence of thromboembolic events was 4% in patients on bevacizumab plus chemotherapy and 2% in patients on chemotherapy alone (Skillings *et al*, 2005). Mortality associated with thromboembolic events was 0.8% in patients on bevacizumab plus chemotherapy compared to 0.4% in patients on chemotherapy alone. Venous thrombosis was reported in 3% of patients on sunitinib, compared to 2% of patients overall, in two trials in renal cell carcinoma. No venous thrombosis was found in patients on placebo in a GIST trial.

Predisposition to thrombosis and bleeding after inhibition of VEGF signalling reflects the multiplicity of actions of VEGF on vascular walls and perhaps on components of the coagulation system. Vascular endothelial growth factor not only stimulates endothelial cell proliferation, but also promotes endothelial cell survival and helps maintain vascular integrity. Inhibition of VEGF could thereby diminish the regenerative capacity of endothelial cells and cause defects that expose pro-coagulant phospholipids on the luminal plasma membrane or underlying matrix, leading to thrombosis or haemorrhage (Kilickap *et al*, 2003). Vascular endothelial growth factor increases production of NO and prostacyclin (PGI_2_, prostaglandin I_2_), suppresses pathways involved in endothelial cell activation, apoptosis, and pro-coagulant changes, and inhibits proliferation of vascular smooth muscle cells (Zachary, 2001). Reduction in NO and PGI_2_ after inhibition of VEGF signalling may predispose to thromboembolic events. Vascular endothelial growth factor inhibition may also increase risk of thrombosis by increasing haematocrit and blood viscosity via overproduction of erythropoietin (Spivak, 2002; Tam *et al*, 2006).

However, endothelial cell defects alone are unlikely to explain life-threatening haemorrhage in patients on anti-VEGF therapy for squamous cell lung cancer and certain other solid tumours. Rather, weakening of the wall of major vessels by tumour erosion, necrosis, cavitation, or other concurrent pathological conditions are likely to play a central role.

### Reversible posterior leukoencephalopathy

Reversible posterior leukoencephalopathy syndrome is a serious but reversible condition characterised by onset of headache, altered mental function, seizures, visual impairment or blindness, usually hypertension, and occipital-parietal subcortical cerebral oedema evident by computed tomography and magnetic resonance imaging. Reversible posterior leukoencephalopathy syndrome has been reported in patients on bevacizumab (Allen *et al*, 2006), sunitinib, or sorafenib (Govindarajan *et al*, 2006), and has been found after chemotherapy alone (Connolly *et al*, 2007). Because RPLS is attributed to hypertensive encephalopathy and endothelial dysfunction leading to breakdown of the blood-brain barrier, focal cerebral oedema, or vasospasm, inhibition of VEGF signalling is implicated in the pathophysiology, but the syndrome often has other contributing factors and has not yet been replicated after VEGF inhibition in preclinical models.

### Cardiac impairment

Impaired cardiac function has been observed in some patients on sunitinib or sorafenib. Left ventricular ejection fraction was below the lower limit of normal in 15% of patients receiving sunitinib for renal cell carcinoma, and 11% of patients on sunitinib required treatment for low ventricular ejection fraction in a study of GIST. Cardiac ischaemia or infarction was reported in 2.9% of patients on sorafenib in a randomised phase III trial in advanced renal cell carcinoma (Kane *et al*, 2006; Escudier *et al*, 2007). VEGF inhibition by systemic administration of soluble ectodomain of VEGFR-1 for 2 weeks resulted in a 32% reduction in cardiac output, but no change in myocardial vascularity, heart rate, left ventricular ejection fraction, or left ventricular fractional shortening in unanaesthetised adult mice (Kamba *et al*, 2006).

### Endocrine dysfunction

Clinical or laboratory evidence of hypothyroidism has been found in a significant proportion of patients on sunitinib for metastatic renal cell carcinoma or GIST and may contribute to fatigue in these patients (Chow and Eckhardt, 2007). Hypothyroidism has also been seen after bevacizumab. Thyroid function could be impaired by regression of capillaries around thyroid follicles. Inhibition of VEGF signalling in adult mice for 1–3 weeks resulted in regression of more than half of the capillaries in the thyroid (Kamba *et al*, 2006). Fenestrated capillaries also regressed in the pituitary, adrenal cortex, and pancreatic islets (Inoue *et al*, 2002; Lammert *et al*, 2003; Kamba *et al*, 2006). Inhibition of VEGF receptor signalling for 3 weeks was accompanied by elevation of blood TSH (Figure 3G), presumably from impairment of the thyroid-hypothalamic feedback loop (Kamba *et al*, 2006).

Mice treated with either of two different VEGF inhibitors had improved glucose handling, despite reduction in vascularisation of pancreatic islets (Kamba *et al*, 2006). Facilitation of glucose handling is paradoxical, because loss of capillaries in pancreatic islets would be expected to impair glucose sensing. It also contrasts with observations of defective glucose sensing found after targeted disruption of VEGF signalling in pancreatic islets of genetically altered mice (Inoue *et al*, 2002; Lammert *et al*, 2003). Therefore, improved glucose handling after systemic inhibition of VEGF signalling probably involves mechanisms other than reduction in islet vascularisation. Consistent changes in glucose handling have not been reported in clinical studies of VEGF inhibitors in cancer patients.

## CONCLUSIONS

A growing number of drugs that inhibit VEGF signalling are being used in the treatment of cancer, commonly in combination with chemotherapy. The efficacy of these agents raises hope for patients with otherwise unresponsive tumours. The agents are generally well tolerated, but may be accompanied by distinct adverse effects, including hypertension and proteinuria. Although usually manageable by conventional medical approaches, side effects of VEGF inhibitors tend not to include alopecia, myelosuppression, or neutropenia found with conventional chemotherapy. Instead, most are downstream effects of suppression of VEGF signalling in endothelial cells of normal organs. Among these are endothelial cell dysfunction and regression of fenestrated capillaries. Better understanding of the underlying mechanism of adverse effects, optimal dosing, and ways of monitoring drug actions is becoming increasingly important as VEGF inhibitors are used more widely and applied earlier in disease, in younger patients, and with longer duration.
